# AI-assisted consent in paediatric medicine: ethical implications of using large language models to support decision-making

**DOI:** 10.1136/jme-2024-110624

**Published:** 2025-08-06

**Authors:** Jemima Winifred Allen, Brian David Earp, Dominic Wilkinson

**Affiliations:** 1 Department of Paediatrics, Monash University Faculty of Medicine Nursing and Health Sciences, Melbourne, Victoria, Australia; 2 Uehiro Oxford Institute, University of Oxford, Oxford, UK; 3 Centre for Biomedical Ethics, National University of Singapore Yong Loo Lin School of Medicine, Singapore; 4 Newborn Care, Oxford University Hospitals NHS Foundation Trust, Oxford, UK; 5 Murdoch Children’s Research Institute, Melbourne, Victoria, Australia

## Abstract

Obtaining informed consent in paediatrics is an essential yet ethically complex aspect of clinical practice. Children have varying levels of autonomy and understanding based on their age and developmental maturity, with parents traditionally playing a central role in decision-making. However, there is increasing recognition of children’s evolving capacities and their right to be involved in care decisions, raising questions about facilitating meaningful consent, or at least assent, in complex medical situations.

Large language models (LLMs) may offer a partial solution to these challenges. These generative artificial intelligence (AI) systems can provide interactive, age-appropriate explanations of medical procedures, risks and outcomes tailored to each child’s comprehension level. LLMs could be designed to adapt their responses to young patients’ cognitive and emotional needs while supporting parents with clear, accessible medical information.

This paper examines the ethical implications of using LLMs in paediatric consent, focusing on balancing autonomy promotion with protecting children’s best interests. We explore how LLMs could be used to empower children to express preferences, mediate family disputes and facilitate informed consent. However, important concerns arise: Can LLMs adequately support developing autonomy? Might they exert undue influence or worsen conflicts between family members and healthcare providers?

We conclude that while LLMs could enhance paediatric consent processes with appropriate safeguards and careful integration into clinical practice, their implementation must be approached cautiously. These systems should complement rather than replace the essential human elements of empathy, judgement and trust in paediatric consent.

## Introduction

The process of obtaining valid authorisation for medical treatment presents challenges in paediatric settings. Unlike adult medicine, where informed consent is grounded in patient autonomy and presumed personal decision-making authority, paediatric healthcare involves a complex interplay between parental responsibilities and authority, children’s evolving capabilities and healthcare providers’ ethical obligations. This complexity requires balancing due respect for all parties concerned, determining appropriate decision-maker(s) and promoting the child’s best interests.^1 2^

In medical decision-making involving children (legally understood as persons under the age of 18 in many jurisdictions), the prevailing moral factor is widely seen as the preservation or promotion of the welfare-based interests of the child. Although these interests *include* giving weight to children’s preferences and decisions in accordance with—and to promote—their developing autonomy, it is commonly held that those with caretaking authority for children must usually be given final say about their medical treatment.^3–6^ In the UK context, for instance, under the Children Act 1989, those with parental responsibility hold primary authority to make medical decisions for children lacking capacity (ie, sufficient understanding and/or maturity to validly authorise a particular medical decision for oneself), while healthcare practitioners must act in the child’s best interests and provide appropriate medical advice. This framework of shared decision-making authority poses challenges shared with other forms of surrogate decision-making, such as aligning decisions with the patient’s best interests,^7^ while also introducing distinctive paediatric challenges like coordinating multiple decision-makers and potentially complex family dynamics.^8^ When disagreements arise between families and healthcare providers, courts serve as the ultimate arbiter. This framework ensures parental decision-making authority is balanced with safeguards to protect children’s welfare.

Parental decision-making on behalf of minor children occupies a unique ethical space, distinct from both adult self-determination^1^ and other forms of proxy decision-making (eg, medical decision-making on behalf of formerly autonomous persons).^9–11^ While young children may have well-considered wishes and preferences, and may be able to give ethically valid consent to some sorts of interventions or procedures,^12^ they may not yet have developed sufficiently stable values, life goals or capacities around evaluating long-term benefits and risks to be able to personally authorise some complex medical decisions.^13 14^ In such cases, parents or legal guardians (hereafter ‘parents’) must make other-regarding decisions from a ‘second-person epistemic standing’—they can observe and interpret their child’s needs and interests, but cannot access the kind of first-person knowledge that gives adult patients clear authority over their own medical decisions.^1^

Beyond establishing decision-making authority, the law imposes specific duties on healthcare practitioners regarding information provision. Healthcare providers have a fundamental duty to adequately inform patients (and/or their legal decision-makers) of the benefits, risks and alternative treatments available. This duty, established in the UK in cases such as Montgomery v Lanarkshire Health Board (2015), requires that patients be given information in a way that enables them to make informed decisions about their care. In paediatric contexts, this duty extends to providing age-appropriate information to children capable of understanding, while ensuring parents or guardians receive comprehensive information necessary for surrogate decision-making. While it is outside the scope of this particular paper to discuss the exact information requirements for adequate disclosure in LLM-supported consent to fulfil the material risk standard of informed consent, this issue is discussed in more detail elsewhere.^15^

Recent decades have seen increasing recognition that decision-making capacity develops progressively,^16^ and involving children in healthcare decisions can both respect them as moral agents and lead to better health outcomes.^2 3^ Children can often meaningfully participate through providing assent—their uncoerced agreement after receiving developmentally appropriate explanations^1^—even when they cannot provide legally valid consent. That being said, in certain jurisdictions, young people who demonstrate sufficient understanding and maturity (referred to as *Gillick competence* in England and Wales law) may be viewed as having the capacity to provide their own autonomous consent to medical treatment.^17^ (However, this ruling primarily establishes a right to consent rather than an absolute right to refuse, and parents or the courts may override a mature minor’s refusal if the treatment is deemed to be in the child’s best interests.)

Despite this progress, significant practical and communication barriers persist in paediatric decision-making. These include formal medical settings inhibiting children’s expression of questions and concerns, time-constrained clinic visits, difficulties in processing and retaining complex medical information and challenges in coordinating multiple decision-makers.^18–21^ These barriers can particularly impact cases involving major interventions,^22^ where effective communication is crucial for treatment adherence^23^ and outcomes.^3^

Generative artificial intelligence (AI), specifically LLMs, could offer promising solutions to some of these challenges, as illustrated by the hypothetical case in box 1.

Having grown up in a digital age, some children may find it less intimidating to interact with an AI-based tool than to participate in some in-person discussions, particularly for sensitive issues.^24^ Early studies suggest LLMs can enhance medical communication through interactive, personalised explanations that complement traditional consent processes.^25 26^ These systems show particular promise in supporting children’s emotional expression^27^ and adolescent mental health engagement.^28^

Similar interactive consent tools for paediatric clinical trials demonstrate improved comprehension among both parents and children,^21^ reduced parental anxiety^29^ and increased satisfaction with the consent process.^30^ The potential integration of LLMs with multimodal capabilities could further enhance accessibility and engagement.^31^

There is a further question as to whether LLMs may be capable of replacing certain more specialised tasks in the consent process (such as assessing the decision-making capacity/Gillick competence of the child).^i^ However, for the purposes of this discussion, we will focus on the use of LLMs as aids in consent-related communication of information.

Previously, we have explored some of the ethical implications of LLM-supported^ii^ consent for medical procedures,^15^ medical research^32^ and biobanking^33^ (table 1). However, introducing AI systems into paediatric consent processes raises distinctive ethical questions. This paper examines how to ensure that LLMs enhance rather than undermine the co-fiduciary relationship between parents and providers, what safeguards are needed to promote appropriate shared decision-making while respecting parental authority and how such systems should handle disagreements between children, parents and healthcare providers. The integration of LLMs into paediatric consent processes must align with existing legal requirements for informed consent, particularly the duty to provide adequate information about benefits, risks and alternatives.

We begin by outlining practical approaches to LLM-supported consent based on children’s age and decision-making capacity. We then explore key ethical considerations including: the role of LLMs in cases where children refuse treatment; managing parent-child disagreements and maintaining appropriate boundaries around information sharing and confidentiality.

### Approach To Llm-Supported Consent For Paediatric Patients

**Figure 1:**
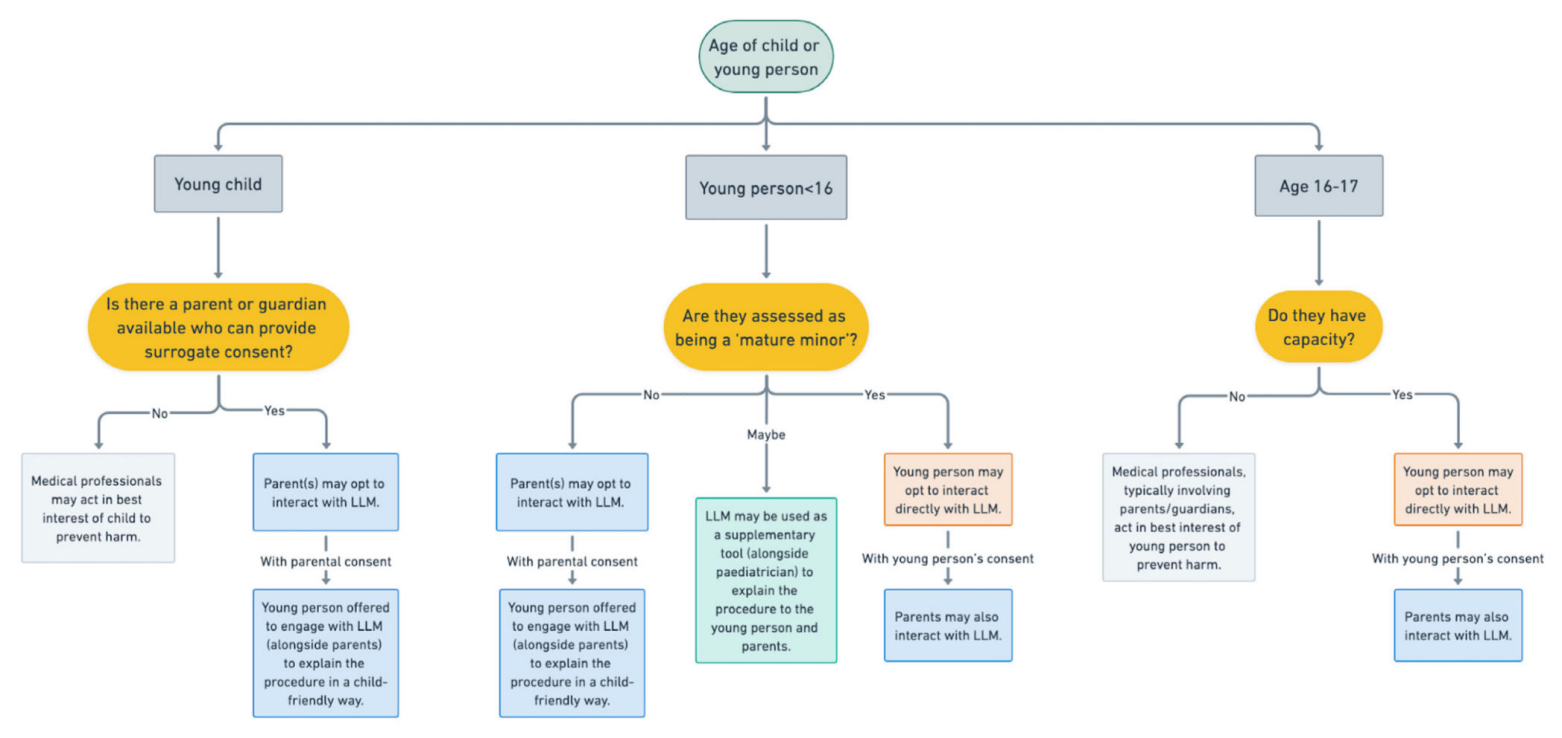
LLM-supported consent pathway for paediatric patients based on age and capacity. This model draws on the ethical and legal framework that applies in England and Wales;^17^ however, it is likely that this model can be adapted to other jurisdictions that recognise categories of mature minors and older adolescents. (In the case of a young person aged >16 years who lacks capacity, it is highly likely that parents are going to be involved (as part of a best interests decision-making process), and it would still be appropriate to offer information via LLM even if parental permission is not formally sought in such cases). LLM, large language model.

Children’s ability to understand medical treatment evolves with age and cognitive maturity. We propose three approaches to LLM-supported consent aligned with established legal and ethical frameworks for paediatric decision-making capacity (figure 1).

Before implementing any LLM-supported consent system, healthcare providers must first obtain appropriate parental authorisation for children to interact with these digital platforms. For young people aged under 16 years, parental consent is required for LLM access, with parents and children potentially using the system together (particularly for younger children). A young person with Gillick competence (or mature minor) status may independently decide whether to involve parents in their LLM consent interactions.

### Young children (not Gillick competent)

For young children or infants, medical decision-making authority rests with parents or guardians, with courts providing authorisation when needed for disputed treatment.^17^ In England and Wales, the Family Division of the High Court has jurisdiction over healthcare decisions for children, particularly in cases involving serious medical treatment where there are disputes between families and healthcare providers.

When LLM systems encounter disagreements between children, parents and healthcare providers, they should be designed to document the nature and extent of disagreements and automatically flag cases requiring potential court involvement. However, LLMs should not attempt to resolve disputes independently.

LLMs can support parental decision-making by providing accessible, tailored medical information to parents, particularly for those facing barriers to in-person consultation. These systems complement, rather than replace, direct healthcare provider communication.

LLMs can also facilitate children’s meaningful participation through age-appropriate explanations that are more readable than traditional consent documents (box 2).^21^ Features like customisable avatars and spoken explanations can make the experience more engaging and accessible for children with varying abilities.^31 34^

### Young persons aged <16 years (Gillick competence)

While legal frameworks vary internationally, many jurisdictions recognise that some young people under 16 can demonstrate sufficient maturity to make independent healthcare decisions, known as ‘Gillick competence’ in England and Wales,^17^ or ‘mature minor’ status elsewhere.

This raises complex ethical questions about how LLMs should interact with young people whose decision-making capacity is still developing. While clinicians retain responsibility for capacity assessment, we recommend LLMs support the consent process through tailored information provision that helps young people develop and demonstrate their capacity for informed decision-making.

For those deemed capable of independent decisions, LLMs must protect developing autonomy while still supporting family involvement when desired. This includes maintaining confidentiality when requested and avoiding undue privileging of parental perspectives. Even when young people are deemed capable of independent decision-making, many choose to involve their parents in medical decisions. LLMs could facilitate these family discussions by providing consistent information to all parties, helping articulate different perspectives and concerns and supporting constructive dialogue between family members.

When young people are not deemed capable of independent decision-making, LLMs (if deployed in paediatric consent processes) could follow the same implementation approach as outlined in *‘Young children or infants (not Gillick competent)*’ section. This involves providing age-appropriate explanations, helping them articulate their views and preferences and supporting their participation in discussions with parents and clinicians. The LLM should also document their input as part of the decision-making process.

### Young persons aged 16–17 years (presumed capacity)

The legal frameworks governing medical decision-making capacity for perons aged 16–17 years vary internationally (table 2). The ethical principles we explore are relevant across jurisdictions that recognise increasing decisional authority in late adolescence.

Young people aged 16–17 years with presumed capacity may provide their own consent for LLM access and must consent to any parental interaction with the LLM system.

For this group, LLMs should approach users as presumptively capable decision-makers, providing adult-level information while maintaining flexibility to adjust explanations if needed. The systems must navigate the unique challenge that these young people often remain practically dependent on parents while having legal authority for medical decisions.

Systems require robust authentication and clear protocols for managing information sharing, with parental involvement remaining at the young person’s discretion (rather than assumed or required). If the young person is assessed to lack capacity in specific circumstances, parental authorisation may be sought (eg, for non-emergency situations where parents are available).^35^ Here too, the court (in this case the Court of Protection) has a role in cases of dispute or uncertainty.

### Refusal and Persuasion

One of the more ethically complex situations in paediatric medicine occurs when a young person refuses recommended treatment. One possibility is to programme LLMs to encourage paediatric patients to reconsider their decisions in situations where refusing treatment could lead to negative health outcomes. However, this raises a critical question for LLM design: Should these systems attempt to persuade young people to accept treatments deemed to be in their best interests?

Understanding this issue requires distinguishing between three forms of influence: persuasion, which involves presenting reasons or arguments that appeal to a person’s rational decision-making capacity; coercion, which uses threats or force that override autonomous choice and manipulation, which neither directly threatens nor purely reasons but rather influences decisions by managing information or exploiting psychological biases to serve the manipulator’s ends rather than supporting autonomous choice.^36^

Crucially, a temporal distinction should be made between persuasion that occurs during the informed consent process versus prompting reconsideration after a decision has been made. In current practice, healthcare providers engage in rational persuasion (ie, explaining treatment benefits and risks, addressing specific concerns and encouraging reconsideration through reasoned dialogue).^37^ However, once a competent adult patient makes an informed decision, current ethical practice does not typically involve encouraging reconsideration, as this would risk undermining patient autonomy.

The question of where AI-prompted reconsideration would sit within the spectrum of persuasion and coercion is particularly complex in paediatric contexts. For young people with established decision-making capacity (ie, Gillick competent or aged 16–17 years), prompting reconsideration after they have made an informed refusal may cross from acceptable persuasion into problematic coercion, especially given the power dynamics inherent in medical settings and the potential for repeated automated prompting. This differs qualitatively from the ongoing dialogue model used with adult patients, where reconsideration typically emerges through patient-initiated questions or changed circumstances rather than provider-initiated pressure.

In paediatrics, the boundary between persuasion and coercion is particularly fine.^38^ On one hand, paediatricians have a duty to ensure that children receive the treatments that are in their best interests, even if the child is initially hesitant. On the other hand, coercing a young person into treatment can undermine their autonomy and trust in the healthcare system, particularly if they are Gillick competent or approaching the age of majority.

While this approach aims to protect health outcomes through transparent communication, implementing persuasive capabilities in LLMs raises distinct ethical concerns that go beyond human-to-human interaction. Recent research demonstrates that personalised messages crafted by AI systems can exhibit significantly more influence than non-personalised messages across different domains.^39^ Unlike human conversation which adapts to social cues, LLMs risk exerting undue influence through persistent messaging that could constitute manipulation, particularly through their ability to engage in reciprocal exchanges and adjust manipulation strategies based on user input in real time.^40 41^

The risk of crossing from persuasion into coercion is heightened when LLMs are programmed to repeatedly prompt reconsideration of refusal decisions. Unlike the time-restricted nature of human clinical encounters, AI systems could theoretically engage in unlimited attempts to change a patient’s mind, potentially wearing down resistance through persistence rather than reason. This pattern of repeated prompting after a decision has been made would likely constitute coercion rather than legitimate persuasion.

Second, the impact of AI-driven persuasion may differ across age groups in complex ways. Overall, young people tend to exhibit greater acceptance of and trust in AI systems compared with adults, who tend to be more sceptical and resistant to AI persuasion attempts.^42^ Younger children tend to anthropomorphise digital agents^43^ and form para-social relationships with conversational AI,^44^ while their limited understanding of persuasive intent may make them especially vulnerable to automated influence. Pre-adolescents, despite emerging critical thinking abilities, show particular susceptibility to targeted engagement techniques through their increased digital media use.^45^ Adolescents present a complex picture: while more technically literate, they remain vulnerable to AI-driven nudging.^46^ Older children also tend to engage with AI systems independently without adult supervision, while the systems themselves typically lack appropriate child-friendly safeguards.^47^ Each age group thus requires specific safeguards in LLM design, including age-appropriate verification systems, structured decision-making supports and monitoring for emotional manipulation that is sensitive to the socio-relational context of the LLM use.^48^

A third crucial challenge stems from LLMs’ limited contextual understanding and relative lack of emotional intelligence,^49^ which could inadvertently amplify rather than alleviate anxious parents’ concerns (eg, through repeated interactions where the LLM continues providing detailed information without appropriately recognising or responding to parental anxiety). This limitation is particularly significant in paediatric contexts, where family relationships and emotional states play crucial roles in decision-making.

These concerns raise a fundamental question: Do the potential benefits of LLM-supported consent justify the risks, particularly given the vulnerability of paediatric populations?

The case for using LLMs despite these concerns rests on three key considerations. First, current paediatric consent processes suffer from persistent communication barriers, time constraints and information asymmetries that compromise decision-making quality.^18–21^ Second, the risks outlined above are design challenges that can potentially be mitigated through implementation strategies, rather than inevitable consequences of LLMs. Finally, existing suboptimal consent processes also carry ethical costs. When children and families lack adequate understanding of medical decisions, or when communication barriers prevent meaningful participation, autonomous decision-making is already compromised. Therefore, LLMs, when properly designed and implemented, may actually enhance autonomy by providing more accessible and comprehensive information than traditional consent processes.

Given these considerations, we propose that LLMs should be implemented under specific conditions and with appropriate safeguards to mitigate these risks (box 3).

The ultimate role of LLMs should remain supportive rather than decisive, focusing on documentation and understanding while healthcare providers maintain responsibility for managing cases where a young person refuses potentially beneficial treatment, including determining when legal intervention may be necessary.

### The Role of the Parent(s)

While medical decision-making in paediatrics primarily concerns the child’s well-being, it typically involves parents or guardians as key decision-makers. As noted, a significant practical challenge is that often only one parent can attend medical appointments or engage directly with healthcare providers, potentially leading to information asymmetries between parents and complications in shared decision-making.^18^

LLMs could help address this challenge by providing a platform through which both parents can access medical information and engage with the consent process, regardless of work schedules or other practical constraints. For example, both parents could independently review detailed treatment information, ask questions and document their concerns through the LLM interface.^26^ Reviewing these interaction transcripts could also offer important insights for healthcare professionals in understanding family preferences and concerns.

This is particularly relevant given current legal frameworks, where it is generally sufficient for one person with parental responsibility to give permission for medical treatment.^iii^ While this approach has practical benefits in avoiding treatment delays, it can lead to one parent having disproportionate influence on decisions. In cases requiring both parents’ agreement, such as non-therapeutic circumcision,^50^ LLMs could ensure equal access to medical information while documenting areas of agreement and disagreement—not replacing but supporting human and legal processes for resolving conflicts. Additionally, by allowing both parents to engage with the LLM independently or together, LLMs could promote fairness by ensuring that both parents feel equally involved and informed, potentially reducing the risk of one parent feeling excluded from crucial decisions about their child’s healthcare.

However, it would be misleading to characterise LLMs as strictly ‘neutral’ or ‘impartial’ mediators—many AI systems embed certain values, assumptions and biases in their design and operation.^51 52^ Rather than claiming strict neutrality, the value of LLMs lies in their potential to facilitate more structured information-sharing and documentation of different perspectives in cases of disagreement between family members or between families and healthcare providers.

Finally, this technology-mediated approach to parental involvement carries risks. While LLMs might help some families reach shared understanding, they could potentially amplify existing conflicts if parents arrive at opposing interpretations of the information provided.^1^ This raises important empirical questions for future research about how LLM-supported consent processes affect family dynamics and decision-making—do they tend to facilitate agreement or exacerbate disagreement? How do different families engage with and interpret information from these systems?^53^

### Building Trust And Maintaining Professional Responsibility

Incorporating LLMs into paediatric consent requires careful management of confidentiality and clear delineation of professional responsibilities.^17^ Studies highlight particular privacy concerns around digital health communication with young people, especially regarding confidentiality of sensitive disclosures, data security and potential unauthorised access by parents or peers.^24^ Thus, the AI platform should be designed to clearly communicate how information will be used, who will have access to it and when information might be shared.

To this end, LLM-supported consent systems should begin with a disclaimer about paediatrician access to digital consent interactions. This approach aligns with GMC guidance on information disclosure.^17^ Research emphasises such transparency in LLM-supported consent helps maintain trust and open engagement.^26^ If users raise questions or reveal information beyond the consent process, the LLM should refer them to speak directly with their paediatrician.

When young people request that certain details not be shared with parents, the response should align with their established decision-making capacity. For Gillick-competent patients or those aged over 16 years, the system should respect their confidentiality preferences by restricting parental access to information, while maintaining complete records for the healthcare team. Younger patients should be prompted to discuss confidentiality concerns with their paediatrician directly, while also alerting the healthcare team to review the interaction.

While LLMs offer efficient information sharing, some families might feel that their concerns are not being given adequate human attention if asked to interact with an LLM. In particular, clinicians may inappropriately outsource consent discussions entirely to LLMs. Rather than replacing human interactions, LLMs should augment clinicians’ capabilities by handling routine information delivery, thereby freeing clinicians to focus on areas where human judgement and empathy are more crucial, such as addressing family concerns, assessing capacity and managing complex ethical discussions. Therefore, any model for integrating LLM-supported consent should include mandatory review of consent interactions by a human clinician, as well as follow-up with a paediatrician.

Ultimately, the overall responsibility for valid consent remains with healthcare professionals.^17^ Thus, LLMs may be incorporated between ongoing consultations with a paediatrician. To balance thorough oversight with clinical time constraints,^20^ LLMs should also be programmed to automatically identify potential ‘red flag’ concerns. The system should be designed to flag explicit concerns (such as expressed anxiety, confusion or reluctance), leveraging the relative privacy of LLM interactions which may facilitate more open disclosure than face-to-face discussions.^24 27^ The system should also monitor for indirect cues, such as hesitant responses, repeated questioning about the same risks or safeguarding concerns indicating potential abuse or coercion (eg, reluctance to engage under certain circumstances, requests to delete or modify previous responses after family consultation, parent refusing to allow child private interaction with the LLM despite age-appropriate capability). While these features might not individually signal problems, their cumulative presence or pattern over multiple interactions may indicate concerns warranting paediatrician review. The LLM should therefore track both acute red flags and subtle patterns that emerge across the consent process.

In addition, such automated monitoring should be designed to account for diverse communication patterns across cultural contexts.^52^ Therefore, the proposed flagging systems should be rigorously tested across diverse populations to avoid perpetuating healthcare disparities.^51^

## Conclusion

The integration of LLMs into paediatric consent processes offers promising opportunities to enhance communication and understanding in medical decision-making. These systems could help address persistent challenges by providing age-appropriate explanations, ensuring consistent information access for families and facilitating better documentation of consent processes.

However, the ethical justification for their use requires demonstrating that potential benefits outweigh the risks, particularly around automated persuasion, confidentiality and the preservation of human judgement in medical decisions (especially in nuanced areas like capacity/competence assessment and treatment refusal). Thus, we propose that LLMs should serve as supplementary tools within a framework that maintains healthcare provider oversight, adapts to children’s developmental stages and supports rather than replaces family communication. Success will require clear protocols for identifying concerns, managing confidentiality and documenting decisions.

The role of LLMs should remain supportive—enhancing communication and documentation while preserving the essential human elements of paediatric healthcare. Healthcare providers must retain ultimate responsibility for ensuring valid consent. Future empirical research will be crucial to understand how families engage with these systems and their impact on decision-making outcomes, guiding refinements to ensure LLMs effectively support more informed and inclusive consent processes while maintaining necessary protections.

## Figures and Tables

**Table 1:** Generic benefits and risks of LLM-supported consent in medicine^15^

Benefits	Risks
▸ Improved communication: LLMs could be designed to deploy a range of strategies to facilitate improved communication of complex medical information, making it easier for users to understand, including: – Simplified explanations – Translation features in user’s native language – Visual aids and diagrams – Analogies and examples – Consent interaction reports (which provide a summary of the key information for patients to review) ▸ Personalised and interactive responses: LLMs could tailor explanations to the user’s specific questions or concerns. ▸ Enhanced accessibility: LLMs provide convenient access to medical information outside clinical settings, at a time and place of the user’s convenience, minimising the need for in-person consultations and allowing users to have multiple consent interactions without time constraints or social pressures. ▸ Enhanced patient engagement: patients may spend more time in consent discussions with AI systems than with human clinicians, potentially supporting patients’ understanding of relevant information.^54^ ▸ Reduced variability in information: LLMs offer standardised information, minimising the risks of inconsistent or incomplete information often seen with human consent takers. ▸ Efficiency in clinical workflows: LLMs could expedite the consent process, allowing clinicians more time for complex cases. ▸ Documentation and legal protection: interactions with LLMs should be documented, ensuring that the information conveyed is traceable for legal or review purposes.	▸ Misinformation and hallucinations: LLMs may generate incorrect or misleading information (hallucinations), which could compromise patient understanding and decision-making if not carefully monitored. ▸ Trust and empathy issues: patients may struggle to trust AI systems for sensitive medical decisions, especially given Al’s lack of genuine (ie, felt rather than simulated) empathy and human connection. ▸ Lack of human sensitivity: Al may fail to recognise nuanced emotional needs, potentially leading to impersonal interactions. ▸ Responsibility and accountability concerns: delegating part of the consent process to Al may raise questions regarding ultimate responsibility, as LLMs should not be used to assess or be held accountable for the quality of consent provided. ▸ De-skilling and over-reliance on Al: doctors may simply accept the consent obtained by the LLM, without thoroughly checking that the process is valid. ▸ Privacy and data security: patient data entered into Al systems introduces additional risks to privacy, requiring stringent data protection measures. ▸ Click-through consent: digital interfaces may lead to passive engagement in the consent process, where patients bypass important information. However, LLMs can incorporate interactive elements (eg, comprehension questions, follow-up queries and mandatory engagement checkpoints) to ensure meaningful participation in the consent process. ▸ Environmental and sustaihnability concerns: large-scale deployment of LLMs raises significant environmental concerns through high energy consumption, carbon emissions and substantial water usage for data centre cooling.
AI, artificial intelligence; LLM, large language model	

AI, artificial intelligence; LLM, large language model.

**Table 2:** Legal standards for medical decision-making capacity in young people across jurisdictions

Jurisdiction	Default legal age for consent	Mature minor/Gillick competence
England and Wales	16+	No set age; assessed case-by-case for those under 16 (Gillick competence).^17^
Scotland	16+	Under 16: assessed for understanding and maturity, similar to Gillick competence.^55^
USA	18+ (varies by state)	Mature minor doctrine generally applies from 14+, depending on state.^56^
Australia	16+ (varies by state)	Mature minor doctrine generally from 14+; assessed case-by-case in all states; generally determined by young person demonstrating sufficient maturity to comprehend information handling and its consequences.^57^
Singapore	21 +	No statutory provision; case-by-case assessment for minors over 16 or 18 based on understanding and maturity (Gillick principles not formally recognised).^58^

## Data Availability

No data are available.

## Box 2 Comparison of ChatGPT-generated explanation for an endoscopy procedure based on the user’s age (eg, a child aged 8 years vs a child aged 12 years).^i^

**For a child aged 8 years:**

Sure! I can explain what an endoscopy is in a simple way.

An endoscopy is a special way that doctors look inside your body to see what’s going on, especially in places like your tummy (stomach) or throat.

Here’s how it works:

▸ The doctor uses a thin, bendy tube called an endoscope.
▸ This tube has a tiny camera and a light on the end, kind of like a flashlight!
▸ The doctor gently puts the tube into your mouth (or sometimes another place, depending on what needs checking).
▸ The camera sends pictures to a screen, so the doctor can see inside you without needing to do any cutting!

So in short:

▸ It helps doctors see what’s happening inside your body.
▸ It uses a small camera on a tube.
▸ And you might get sleepy so it doesn’t hurt at all.

**For a child aged 12 years:**

Sure thing—I can definitely explain what an endoscopy is in a simple way.

What is an endoscopy?

An endoscopy is a medical test that doctors use to look inside your body, especially in places like your stomach, throat or intestines.

They use a special tool called an endoscope. It’s like a long, thin, bendy tube with a tiny camera and light on the end. This tool helps the doctor see what’s going on inside you without doing surgery.

What happens during it? Here’s how it usually goes:

1. You lie down on a bed in a hospital or clinic.
2. The doctor may give you some medicine to help you relax or even make you sleepy, so you don’t feel much.
3. The endoscope goes into your mouth (or sometimes your bottom, depending on where they need to look).
4. The doctor looks at a screen that shows what the camera sees.
5. When they’re done, they gently take the endoscope out.

Most people don’t feel pain, especially if they’re given relaxing medicine. It might feel a little weird, but it’s quick and safe.

Why do doctors use it? Doctors do an endoscopy to:

▸ See if anything is wrong inside, like an infection or sore.
▸ Check for bleeding or swelling.
▸ Take a small sample (called a biopsy) to test in a lab.

If you’re going to have one, the doctor or nurse will explain everything, and you can always ask questions. Do you want to know what it looks like or why someone your age might need one?

Let me know if you’d like this converted into a printable format or slide layout.

## Footnotes

i All patient scenarios described in this paper are entirely hypothetical and created for illustrative purposes. No actual patient information or identifiable details are included. Ages and names are fictional elements used to demonstrate age-appropriate communication strategies in paediatric consent processes.

i See footnote i.

i While it may be theoretically possible for LLMs to assist with capacity assessment, we set aside this possibility given the nuanced and legally protected nature of such determinations, particularly in sensitive contexts like access to sexual health services or gender-affirming care.

ii The term ‘LLM-supported consent’ is used throughout this paper to emphasise that these systems should enhance and assist existing processes, while maintaining healthcare professionals’ ultimate responsibility for valid consent and preserving essential human elements of empathy, judgement and trust in paediatric decision-making.
